# Development and Evaluation of a Chloramphenicol Hypertonic Ophthalmic Solution

**DOI:** 10.4103/0250-474X.40334

**Published:** 2008

**Authors:** A. V. Jithan, C. Krishna Mohan, M. Vimaladevi

**Affiliations:** Department of Pharmaceutical Sciences, Andhra University, Visakhapatnam - 530 003, India

**Keywords:** Chloramphenicol, stability, solubility, hypertonic solution, cosolvents

## Abstract

Hypertonic ophthalmic solutions are used to treat ocular diseases associated with edema. In this study, we developed a chloramphenicol hypertonic ophthalmic solution. These drops were developed based on the cosolvency and additional dielectric constant concepts. Two different solvents: PEG 300 and glycerol were used as cosolvents. Solubility curves were plotted. Based on the solubility curves, two different solutions were selected. These solutions were evaluated for physical parameters and accelerated stability. The results indicated that chloramphenicol was stable in these formulations. The selected blend of solutions was hypertonic. Thus, the solubility and stability of chloramphenicol was enhanced using a cosolvency technique so as to develop a chloramphenicol hypertonic ophthalmic solution.

Chloramphenicol is the drug of choice in ocular infections because of its high transcorneal penetration and broad spectrum activity, despite its toxic manifestations^1^. Chloramphenicol is available as ophthalmic drops and ointments. In purified water the solubility of chloramphenicol is only 0.25%. Several approaches such as pH adjustment, complexation^2^, micellar solubilization^3^, cosolvency^4^,^5^ were successfully employed to enhance the solubility. The present market and official preparations contain chloramphenicol in water, the pH adjusted with boric acid/borax buffer to enhance to the required solubility (5 mg/ml). Similarly, when the pH is raised to 8.6, its solubility reaches to 1%. However, the stability and activity are much reduced. In the marketed preparations, viscosity promoters are also incorporated so as to enhance the contact time. The negative aspects of these formulations are the stability and the use of phenyl mercuric nitrate (PMN) as preservative. The stability of these formulations is less and PMN has been found to manifest toxic reactions. Thus, the present work was carried out to increase the solubility of chloramphenicol and enhance its stability in ophthalmic drops.

Solubility of non-ionizable substances can be enhanced by the addition of non polar solvents and when mixture of solvents such as PEG 300 are used along with water, the technique is generally termed as cosolvency techinque. Beside solubility, stability is also affected by solvents in a favourable or in a non-favourable direction^6^,^7^. We intended to prepare chloramphenicol ophthalmic solutions with higher soluble drug content using PEG 300 and glycerol as cosolvents because of their higher solubility potential, low toxicity and these are inexpensive. Propylene glycol was not selected as one of the solvents because it must not be used as a solvent for chloramphenicol eye drops or nasal drops because it causes marked burning sensation^8^. Theoretical equations for solubility in water and in binary solvents have been reported in the literature^9^,^10^. Formulation of ophthalmic drops can be made using the solubility curves based on Additional Dielectric Constant (ADC) calculations and these were the physical pharmacy principles employed in the formulation development. The methods, data and discussion involved in the selection of formulations, solubility determinations, methods of preparation, stability, etc. are presented in this manuscript.

## MATERIALS AND METHODS

Chloramphenicol was obtained from M/S Akhil Pharma, Hyderabad. Polyethylene glycol 300, glycerin and benzalkonium chloride were obtained from S.D. Fine Chemicals, Bombay. Methanol was obtained from Ranbaxy Chemicals, Delhi. A UV spectrophotometer, Shimadzu UV-240, Japan was used to analyze the samples. G5 filters from Borosil were used for filtration sterilization of the solutions. An Oswalds Viscometer from Pyrex, Bombay was used to measure the viscosities of the samples. A Clico Digital pH meter was used to determine the pH of the samples. A Laminar Air Flow System from Klenzides, Bombay, was used.

### Standard curve of chloramphenicol:

Chloramphenicol (4 mg) was dissolved in 100 ml distilled water in a volumetric flask to get a 40 μg per ml solution. Aliquots of this solution (1, 2, 3, 4 and 5 ml) were taken in a series of 100 ml volumetric flasks and volume was made up to 100 ml with distilled water to get solutions containing 4, 8, 12, 16 and 20 μg/ml, respectively. The absorbance of these solutions was measured using a UV-Spectrophotometer at 278 nm. Standard readings were taken from triplicates of samples so as to obtain a statistically validated data.

### Solubility of chloramphenicol in solvent blend:

Different blends of the solvents, glycerin, polyethylene glycol 300 and water were prepared (Table 1). Equal amounts of these blends were distributed into 100 ml conical flasks. To each of these blends excess of chloramphenicol was added. The conical flasks were sealed properly through out the experiment to prevent the evaporation of the solvents. These conical flasks were rotated on a rotary shaker for 36 h. Excess of chloramphenicol was added intermittently into the required blends after checking for visual unsaturation. After 36 h of shaking they were stored in dark for 12 h. These solutions were filtered and analyzed for the drug. Then they were stored in the conical flasks at 5° in a refrigerator for 24 h and again filtered to separate out the precipitation. These solutions were analyzed for the drug. The solubilities in the different blends were noted (Table 2). These values were converted to logarithmic scale. Additional dielectric constant (ADC) of the solvent blend was calculated (Table 1). Dielectric properties of mixed solvent system can be approximated as the weighted average of the properties of the pure component. The different volume ratios were converted to weight ratios and additional dielectric constants were determined.

**TABLE 1:** SOLVENT BLEND AND ADDITIONAL DIELECTRIC CONSTANTS

Blend No.	PEG 300		Glycerin		Water weight (mg)	Viscosity (poise)	ADC**
	
	Vol (ml)	Weight (mg)	Vol (ml)	Weight (mg)			
I	5	7.5	10	12.49	85	0.0143	72.589
II	5	7.5	20	24.98	75	0.01824	71.392
III	5	7.5	30	37.47	65	0.02388	68.388
IV	5	7.5	40	49.96	55	0.0397	65.384
V	10	15	10	12.49	80	0.062	70.05
VI	10	15	20	24.98	70	0.017869	62.67
VII	10	15	30	37.47	60	0.024678	61.22
VIII	10	15	40	49.96	50	0.03372	57.1
IX	15	22.5	10	12.49	75	0.043617	67.51
X	15	22.5	20	24.98	65	0.0198	63.05
XI	15	22.5	30	37.47	55	0.028	58.79
XII	15	22.5	40	49.96	45	0.0408	54.73

Proportions of solvent blends and calculation of additional dielectric constants. *PEG 300: polyethylene glycol 300

** ADC: additional dielectric constant

**TABLE 2:** SOLUBILITY OF CHLORAMPHENICOL IN VARIOUS SOLVENT BLENDS

Blend No.	Solubility		Log (solubility)	
		
	AT RT	5°	AT RT	5°
I	7.6	4.4	0.88	0.643
II	8.9	4.5	0.95	0.653
III	19.6	6.3	1.29	0.799
IV	14.76	7.0	1.17	0.85
V	16.5	9.0	1.21	0.954
VI	22.0	-	1.342	-
VII	-	13.4	-	1.127
VIII	26.0	18.0	1.415	1.255
IX	-	12.44	-	1.09
X	24.0	14.3	1.38	1.155
XI	35.0	16.8	1.544	1.225

Solubility of chloramphenicol in various solvent blends at room temperature (RT) and 5° (storage conditions)

Various solubility curves were plotted. In one case, the concentration of polyethylene glycol 300 was held constant while the concentration of glycerin was changed. A graph was plotted with solubilities of chloramphenicol *vs*. the concentration of glycerin. In the second case, the graph was plotted with log (solubility) of chloramphenicol *vs*. additional dielectric constant (ADC) of the solvent blend.

### Formulation of ophthalmic drops:

From the solubility curves two different blends were selected so as to incorporate 4 mg/ml and 5 mg/ml drug in the blend in the solubilized form. These were formulated into two different solution dosage forms MVA1 and MVA2. The two formulations were prepared in bulk, sterile filtered using a G5 filter under aseptic conditions. They were transferred into sterile glass vials under aseptic conditions. Sterile rubber closures saturated with benzalkonium chloride were used in closing the vials. The vials were both amber coloured and plain vials because chloramphenicol is light sensitive and plain coloured vials were used to test the stability under accelerated light conditions. The various parameters of the formulations were viz. viscosity, colour, clarity, pH and drug concentration.

### Stability of the formulations:

The two sterile formulations were subjected to heating (100° for 30 min) and autoclaving (20 min at 15 psig). The vials were also stored under accelerated stability test conditions (37°, 45°, 55°) and various chemical and physical parameters were observed for 5 weeks. A market product was also kept for accelerated stability studies to make a comparative assessment.

## RESULTS AND DISCUSSION

**Fig. 1:**
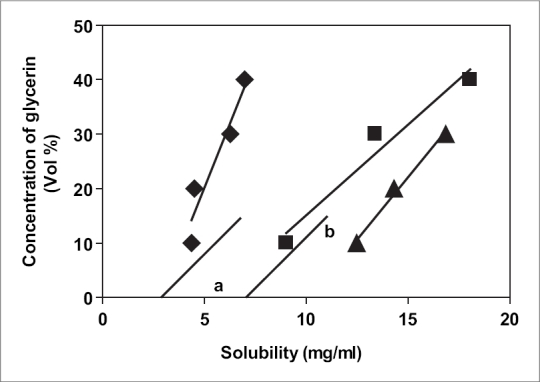
Solubility of chloramphenicol in solvent blend at 5°. Solubility of chloramphenicol in the solvent blend at 5° concentration vs. concentration *vs.* solubility curve 5% PEG (◆), 10% PEG (■) and 15% PEG (▲).

**Fig. 2:**
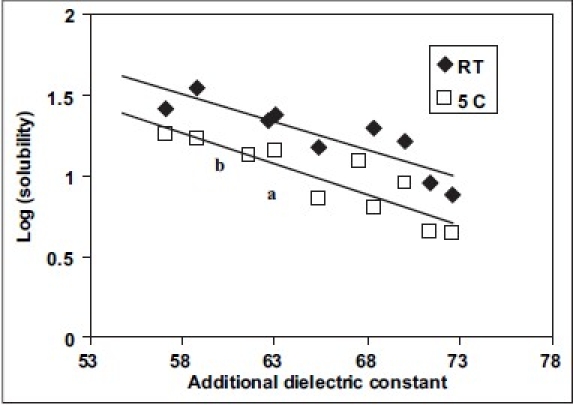
Solubility curves of chloramphenicol in solvent blends. Solubility of chloramphenicol in solvent blends. Plots of log(solubility) *vs.* additional dielectric constant. room temperature (◆) and 5° (□).

**Fig. 3:**
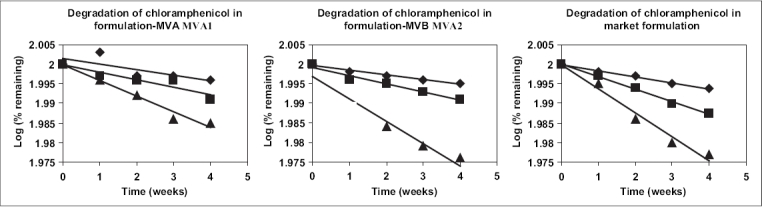
Degradation of chloramphenicol in various formulations. Degradation of chloramphenicol in the selected and the market formulations, 37° (◆), 45° (■) and 55° (▲).

Two different solubility curves as shown in the figs. 1 and 2 were obtained. From the solubility curves two different blends were selected so as to incorporate 4 mg/ml and 5 mg/ml drug in the blend in the solubilized form. Solubility of chloramphenicol at 5° in 10:2:88 blend (formulation MVA1) was determined, fig. 2: point a: solubility = 6.6 mg/ml; fig. 3: point a: Log (solubility) = 0.826 = solubility = 6.7 mg/ml, ADC of the blend = 65. Similarly, solubility of chloramphenicol at 5° in 15:5:80 blend (Formulation MVA2) was determined: fig. 2: point b: solubility = 10.5 mg/ml; fig. 3: point b: Log (solubility) = 1.02 = solubility = 10.6 mg/ml, ADC of the blend = 61. Thus, the blends selected were such that their hold capacity was 167.5% more in formulation MVA1 (drops with 4 mg/ml drug) and 212% more in Formulation MVA2 (drops with 5 mg/ml drug). The compositions of these formulations are as follows: formulation MVA1 (chloramphenicol: 0.4%, PEG 300: 15%, glycerol: 2.5%, benzalkonium chloride: 0.02%, disodium EDTA: 0.0001%, distilled water: upto 100%) and formulation MVA2 (chloramphenicol: 0.5%, PEG 300: 22.5%, glycerol: 12.5%, benzalkonium chloride: 0.02%, disodium EDTA: 0.0001%, distilled water: upto 100%). Both the formulations are to be sterilized by filtration sterilization.

**Fig. 4:**
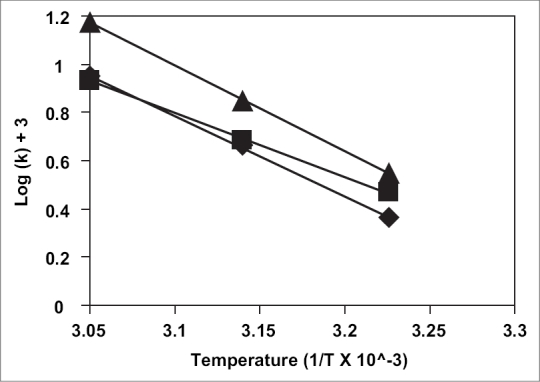
Arrhenius plot for the degradation of chloramphenicol in the prepared formulations. Arrhenius plots for various formulations, formulation MVA1 (◆), formulation MVA2 (■) and the marketed formulation, Chlorocol Eye Drops, Jawa Pharmaceuticals (India) Pvt. Ltd. (▲).

The selected two sterile formulations were subjected to heating (100° for 30 min) and autoclaving (20 min at 15 psig). Upon heating, 3% and 3.2% chloramphenicol degraded in the formulations MVA1 and MVA2, respectively and upon autoclaving 8% and 9% chloramphenicol degraded in MVA1 and MVA2. Further studies yielded physical and chemical stability data. The shelf-life of the selections along with a prescription product available in the market was also determined. Formulation MVA1 was colour-less for all the 5 w at all temperatures and it was clear at the end of 5^th^ w in all temperatures. The pH of 4.25 was retained at 5^th^ week at all temperatures with no change in viscosity during this time. Similar results were obtained with the formulation MVA-2 with the pH staying at 4.45 during all the five weeks of the study. The change in the drug content in the formulations is presented in the fig. 3. Further, Arrhenius plot was plotted (fig. 4) and the shelf-life of Formulation MVA1, Formulation MVA2 and Market Formulation were 19 mo, 14 mo 2 w and 12 mo 3 w, respectively.

To enhance the stability and obtain a desired 0.5% chloramphenicol solution suitable to enter the potential market, several attempts such as pH adjustment, micellar solubilization, cosolvency technique etc., were attempted. However, on many occasions tonicity is compromised. On the other hand, the recent trend on these lines is that more stress has to be laid on the issues like stability, preservatives, sterility rather than tonicity. Atleast it is practically known that human eye can tolerate hypertonic ophthalmic solutions better than known or thought. It has been shown that a range of 0.52 to 2.0% sodium chloride equivalency does not cause marked pain response and that a range of 0.7 to 1.5% should be acceptable to most persons. However, under extreme conditions, preparations as high as 5% sodium chloride are also used. These are used in the treatment of corneal edema. In certain other cases the therapeutic concentration of the drug will necessitate using what might otherwise be considered an unacceptable tonicity. This is the case with sodium sulfacetamide, where the isotonic concentration is about 3.5% but the drug is used in 10 to 30% concentrations. Fortunately, the eye seems to tolerate hypertonic solutions better than hypotonic solutions. Keeping this leverage in mind, we aimed at developing different chloramphenicol ophthalmic drops than those found in the market. The simplest and most common idea that could come to the mind if tonicity is backlogged is cosolvency technique. We performed a literature search if this strategy has been previously mentioned to develop chloramphenicol ophthalmic drops. As mentioned in the introduction, a patent which has been granted to Allergan, used the technique of cosolvency. This is US patent No. 3,702, 364, on the names of Boghosian M and Wilson J. (to Allergan Pharmaceuticals, Nov. 1972). Based on the patent a product was also launched and marketed by Allergan. Chloramphenicol 0.5% ophthalmic solution, which is no longer marketed, was formerly available from Allergan (Chloroptic) and Altana. It contained chloramphenicol, chlorobutanol, polyethylene glycol 300, polyoxyl 40 stearate, sodium hydroxide or hydrochloric acid and purified water. Chloramphenicol ophthalmic solution USP is a sterile solution of chloramphenicol containing not less than 90.0% and not more than 130.0% of the labeled amount of chloramphenicol. It has a pH in the range of 7.0 to 7.5, unless it is unbuffered, when the pH will be between 3.0 and 6.0. Its preparation method has been recently published^11^. In this preparation only polyethylene glycol 300 was used as a cosolvent. However, we aimed at further developing this formulation by incorporating glycerol in the formulation so as to further enhance the stability. However, to begin with the thought of hypertonicity has been there with these formulators. Thus, in this study a chloramphenicol hypertonic ophthalmic solution was prepared.

Frequently a solute is more soluble in a mixture of solvents than in one solvent alone^12^. This phenomenon, as indicated in the introduction, is called co-solvency, and the solvents that in combination increase the solubility of the solute are called co-solvents. For example, approximately 1 g of phenobarbital is soluble in 1000 ml of water, in 10 ml of alcohol, in 40 ml of chloroform, and in 15 ml of ether at 25°. On the other hand 1.5% w/v of phenobarbital is dissolved in 22% alcohol, 40% glycerin and the remaining water (38%). Thus, the aqueous solubility of phenobarbital is increased in cosolvents. We have employed similar strategy in this study. The three solvents selected were glycerol, PEG 400 and water. Two different solubility curves were developed by determining the solubility of chloramphenicol in the different blends of the solvent. A straight line was obtained when log (solubility) was plotted against Additional Dielectric Constant (ADC) of the blend. Similar results are previously found in the literature^12^. Different other mechanisms are also reported. Almost parallel lines were obtained when solubility was plotted against glycerol concentration keeping polyethylene glycol concentration constant. Hence, these solubility curves were utilized to select the blends of solvents for chloramphenicol solution at an optimum concentration of drug as per the dose requirements and stability. Two different formulations were selected based on the solubility curves. Comprehensively, the selected formulations are mentioned in the results.

The above two formulation prepared with the aid of solubility curves had tonicities E = 2 and E = 3.5, respectively. These are hypertonic from conventional formulations where E = 1. That is the reason these could be considered chloramphenicol hypertonic ophthalmic solutions. These are indicated in bacterial infections and bacterial infections accompanied by corneal inflammations where the drug effectively acts against infection and the tonicity helps in reducing the inflammation, oedema due to inflammation and also increases the penetration of the drugs. Disodium EDTA was included in the formulations because it enhances the preservative efficacy of benzalkonium chloride. Thus, in the design of our experimental study the solubility enhancement of chloramphenicol was attempted to develop a stable and suitable ophthalmic drops. The stages involved in the formulation of ophthalmic solution of chloramphenicol are: 1. development of cosolvency system to enhance the solubility of chloramphenicol; 2. preservation; 3. pH adjustment; and 4. sterilization. The stability of the two formulations MVA1 and MVA2 are greater than a commercial sample. Shelf life of these formulations MVA1, MVA2 and market product (Chlorocol Eye Drops, Jawa Pharmaceuticals (India) Pvt. Ltd.) are 19 mo, 14 mo 2 w and 12 mo 3 w, respectively. The variation in the stability of MVA1 and MVA2 may be due to the solvent effects. Thus these can be considered stable formulations. The viscosities of MVA1 and MVA2 are 16.3 and 18.8 cps, respectively. In these viscosity ranges the contact time of a drug can be enhanced. The degradation of the drug on autoclaving was 8% and 9% with the formulations MVA1 and MVA2, respectively. Filteration sterilization using a G5 filter was used to sterilize the formulations. The formulation parameters studied gave satisfactory results. However, clinical tests like contact time, irritatability and pharmacokinetics are to be attempted for thorough product information.
